# 25 years into research with the Méhes Scale, a comprehensive scale of modern dysmorphology

**DOI:** 10.3389/fpsyt.2024.1479156

**Published:** 2024-11-04

**Authors:** Dalma Tényi, Györgyi Csábi, József Janszky, Róbert Herold, Tamás Tényi

**Affiliations:** ^1^ Department of Neurology, Medical School, Clinical Center, University of Pécs, Pécs, Hungary; ^2^ Department of Pediatrics, Medical School, Clinical Center, University of Pécs, Pécs, Hungary; ^3^ Department of Psychiatry and Psychotherapy, Medical School, Clinical Center, University of Pécs, Pécs, Hungary

**Keywords:** minor physical anomalies, Méhes Scale, neurodevelopmental disorders, schizophrenia, epilepsy

## Abstract

It has been recognized that subtle, cosmetically insignificant anomalies tend to occur cumulatively in diseases with neurodevelopmental origin. These visible signs of morphogenesis errors are called minor physical anomalies (MPAs), serving as sensitive external markers of abnormal neurodevelopment. After the introduction of the Waldrop Scale, the studies conducted on MPAs in diseases with neurodevelopmental origin gave conflicting results. It has been debated that this discrepancy can be – at least partly – attributed to the use of the Waldrop Scale. Understanding the need of a comprehensive scale of MPAs that also differentiates according to the time of development, Hungarian pediatrician professor of University of Pécs, Károly Méhes developed a scale with 57 items, the only scale differentiating minor malformations from phenogenetic variants. With the use of the Méhes Scale, our research group has been investigating the role of abnormal neurodevelopment in different neuropsychiatric and neurologic disorders since 1997. 25 years into our research, in this review we summarize the results of our 18 research articles on MPAs in different diseases. We have found an increased number of MPAs, especially in the head and mouth region, in patients with schizophrenia, bipolar disorder, Tourette syndrome, autism and many epilepsy syndromes, fortifying the role of abnormal neurodevelopment in these diseases. Moreover, an increased number of MPAs was detected among the first-degree relatives of patients with schizophrenia and bipolar I disorder, supporting the hypothesis about MPAs being endophenotypic trait markers.

## 1 Introduction

Neurodevelopmental disorders are complex chronic conditions defined by deficits in the domains of motor skills, cognition, behavior and/or communication, appearing on grounds of abnormal central nervous system (CNS) development. The Diagnostic and Statistical Manual of Mental Disorders, 5th Edition, Text Revision: DSM-5-TR categorizes the following conditions under Neurodevelopmental Disorders: autism spectrum disorder, attention deficit hyperactivity disorder (ADHD), intellectual disability, communication disorder, specific learning disorders, motor disorders and tic disorders. The diagnosis of each disorder is based on a constellation of behaviors and symptoms listed in DSM-5-TR (1). Recently it has been debated whether neurodevelopmental disorders are in fact independent entities. It is widely known that different conditions tend to overlap, thus it is rather unusual for a neurodevelopmental disorder to appear on its own (2, 3). Frequent comorbidity can be observed in case of autism spectrum disorder and ADHD (4), moreover, according to DSM-5-TR (1), autism spectrum disorder serves no longer as an exclusion criterion for ADHD as in previous versions. Patients with Fragile X syndrome, a common cause of inherited intellectual disability, are also at risk of autism (5). Children with cerebral palsy may have co-existing ADHD, autism spectrum disorder or intellectual disability (6, 7). Another key defining characteristic is the disease onset, as these disorders generally present in childhood, moreover the cognitive and behavioral dysfunctions do not tend to appear immediately after the insult, but stay latent and become identifiable upon different stages of brain development. For example, in children with mild perinatal asphyxia, cognitive or behavioral abnormalities may become apparent only years after birth and till then, only hardly noticeable, minor neurological signs may be detected. It is also widely known that the symptoms of neurodevelopmental disorders do not always remain constant but may fluctuate, become worse or get better. Additionally, although the symptoms may be significantly improved with either pharmaco- or behavioral therapy long term, neurodevelopmental disorders are incurable disorders to date (2). On grounds of clinical observations in terms of similarities among different neurodevelopmental disorders, it has become questionable whether the strict, behavior based diagnostic approach is valid by all means. Another important aspect is the realization that in addition to the classic, DSM-5-TR defined neurodevelopmental disorders, abnormal CNS maturation plays a role also in other neurologic and psychiatric diseases: for example epilepsy of cortical dysplasia etiology or heterotopic lesions in schizophrenia (8). Growing amounts of scientific evidence suggest the multifactorial nature of neurodevelopmental disorders in terms of etiology, since – among others - chanellopathies, connectopathies, abnormal neurotransmission, genetic defects and epigenetic impact (perinatal asphyxia, vitamin deficiency, infections, toxic effects) may result in abnormal neurodevelopment (9). Many methods are available for the detection of aberrant neurodevelopment, for either clinical or research purposes (10). Careful medical history taking in terms of the pre-, peri- and early postnatal period and the later psychomotor development is highly important, since anomalies in these phases may suggest an underlying maldevelopment. Genetic testing may detect the single, direct cause of the disease (e.g. Fragile X syndrome) or point out genetic variants predisposing to abnormal neurodevelopment. Neuroimaging and postmortem brain studies may also serve as important methods in the detection of the structural background of behavioral and cognitive symptoms. It is evident that examining methods are not scarce, although more of them may not be available in every clinical setting due to technical or financial reasons.

## 2 Minor physical anomalies as trait markers for abnormal neurodevelopment

Central nervous system development begins around the 3. week of gestation, in the very beginning of the so-called embryonic stage of development (3-8. week), during which organogenesis takes place. At the end of the 2^nd^ gestational week, the primitive streak becomes apparent and quickly starts to differentiate into the endo-, meso- and ectoderm (11). The pluripotent cells of the ectoderm begin to produce neuroepithelial cells, forming the neural plate, which invaginates and closes, creating the neural tube by the end of the 3^rd^ gestational week. Around the 5^th^ gestational week, the cranial end of the neural tube begins to swell, and differentiate into the telencephalon (which at the end forms the cerebral cortex, basal ganglia and hippocampus), diencephalon (developing into thalamus and hypothalamus), mesencephalon, metencephalon (later forming the pons and cerebellum) and myelencephalon (developing into the medulla oblongata) (11). At the 5^th^ gestational week, the two important parts of the telencephalon can already be differentiated: the dorsal and the ventral neurogenic zones. The progenitor cells of the dorsal part migrate radially, developing into the cortical excitatory pyramidal neurons till the 20-23^rd^ gestational week. The progenitor cells of the ventral part, on the other hand, start to migrate tangentially, forming the cortical inhibitory neurons, which process continues to go on even postnatally (11, 12).

Since the surface ectoderm (which later forms the skin) and the neuroectoderm differentiate from the same ectodermal tissue early in gestation, it seems reasonable to assume that an insult affecting this vulnerable stage of development may cause both CNS and skin anomalies. In fact, this connection is well-established in phacomatoses (also known as neurocutaneous syndromes), in which one of the main disease characteristic is the co-occurrence of skin and CNS anomalies: café au lait spots, schwannomas and neurofibromas in neurofibromatosis type I and II; facial angiofibromas and cortical tubers in tuberous sclerosis; leptomeningeal angioma and nevus flammeus in Sturge-Weber syndrome (13).

Analogue to the phenomena observed in neurocutaneous disorders, it has been recognized that subtle, cosmetically insignificant (mostly even only hardly recognizable) anomalies, identifiable even in healthy individuals, tend to occur cumulatively in diseases with neurodevelopmental origin. These visible signs of morphogenesis errors are called minor physical anomalies (MPAs), serving as sensitive external markers of abnormal neurodevelopment and may carry major informational value for diagnostic, prognostic and epidemiological purposes (14, 15). They are considered to be results of maldevelopment between the 3^rd^ and 20-23^rd^ gestational week and since they persist into adult life they can be detected on physical examination at any age from neonates to the elderly.

## 3 Scales to measure minor physical anomalies – development of the Méhes Scale

The first comprehensive studies on the abundance of MPAs in diseases with disordered behavior dates back to the 1960s. Goldfarb and Brostein (16) discovered that children with schizophrenia present with a higher number of MPAs. In their pioneering work, Mary Waldrop and Goering (17) detected increased number of minor physical anomalies in hyperactive children using the 18 item long Waldrop Scale, developed based on the work by Goldfarb and Brostein (16). After the introduction of the Waldrop Scale, a great amount of studies were conducted studying the connection between MPAs and neurodevelopmental disorders. Studies on the prevalence of MPAs in patients with hyperactivity (17), schizophrenia (18–24), affective disorders (21, 24) gave conflicting results (25–27). Although the development of the first scale to evaluate MPAs was highly innovatory and gave exceptional insight into the neurodevelopmental background of many disorders, it has been debated that the discrepancy between the studies can be – at least partly – attributed to the use of the Waldrop Scale and the shortcomings thereof. The Waldrop Scale contains only 18 MPAs, while in pediatric literature more than 50 have been listed. In the late 90s, only one year apart, three new scales have been developed. Recognizing the shortness of the Waldrop Scale as its weak point, in 1997, Lane and her coworkers (28) introduced a scale with 62 items for the assessment of dysmorphic features in schizophrenia. A year after that, pointing out its lack of extensiveness and the fact that these 18 item long list originated from results of an unpublished study, Ismail and his coworkers (29) conducted another scale with 41 items to investigate MPA profile in patients with schizophrenia. Besides the low number of items, the Waldrop Scale has been also criticized for not distinguishing between MPAs according to their time of development (30). Based on the report of the International Working Group (31), both Opitz (32) and Méhes (25) urged a clear distinction between morphogenetic events developing during organogenesis (i.e. in the embryonic state) and after organogenesis.

Understanding the need of a complex, comprehensive scale of MPAs that also differentiates according to the time of development, Hungarian pediatrician professor of University of Pécs, Károly Méhes developed a scale with 57 items, the only scale that differentiates minor malformations from phenogenetic variants (**Table 1**). Minor malformations are always abnormal, “all-or-none” type qualitative defects of embryogenesis, arising during organogenesis. On the other hand, developing after organogenesis, phenogenetic variants represent quantitative defects of final morphogenesis, and they can be regarded as exact equivalents of normal anthropometric variants (14, 25, 32). The background of the development of the Méhes Scale, a comprehensive instruction of its use, a detailed description of each anomaly and also, results of early studies on the topological profile of MPAs with the use of the Méhes Scale in childhood malignancies, diabetes, intellectual disability and cerebral palsy has been published in the pioneering work entitled “Informative morphogenetic variants in the newborn” by Méhes in 1988 (14).

**Table 1:** The Méhes Scale.

Minor malformations	Phenogenetic variants
Preauricular tag Preauricular pit Lip pit Bifid uvula Supernumerary nipples Partial syndactily of toes 2-3 Pigmented naevi Café-au-lait spots Hemangioma Sacral hemangioma Prominent occiput Prominent forehead Flat forehead Flat occiput Primitive shape of ears Cup ears Earlobe crease Simian crease Sydney line Single flexion crease on the 5^th^ finger Sole crease Prominent heel Double posterior hear whorl Multiple buccal frenula Furrowed tongue Brushfield spots Fine electric hair Tongue with smooth and rough spots Frontal upwap Lack of earlobe Double anthelix	Small mandible Confluent eyebrows Short palpebral fissures Mongoloid slant Antimongoloid slant Inner epicanthic folds Hypertelorism Asymmetrical size of ears Protruding auricle Low set of ears Soft and pliable ears Abnormal philtrum Large or small oral opening High arched palate Large tongue Short sternum Wide-set nipples Acromial dimples Deep sacral dimple Unusual length of fingers Clinodactily Hallucal abnormality Wide distance between 1^st^ and 2^nd^ toes Nail hypoplasia Dimple on the tuberositas tibiae Dimple on the elbow

Owing to its complexity and comprehensiveness, training is needed to be able to correctly apply the Méhes Scale in terms of adequately determine MPAs. Another limitation of the Scale is that based on the MPA profile it does not determine the kind of insult afftecting the central nervous system (e.g. genetic, vitamin difciency, trauma etc.), further research is needed in this direction.

## 4 25 years into the research on neurodevelopment with the use of the Méhes Scale

Conducted by a trained physician, the thorough physical examination according to the Méhes Scale may be carried out in a couple of minutes. As the examination requires no special equipment (in case of certain items only caliper and tape to improve objectivity), it serves as an effective, sensitive, fast and low-cost method for not only research purposes but in everyday clinical practice to identify patients with a possible underlying disorder with neurodevelopmental origin.

With the use of the Méhes Scale, our research group has been investigating the role of abnormal neurodevelopment as a contributing etiological factor in different neuropsychiatric disorders. Starting off with the investigation of patients with schizophrenia, we published our first results in 1997. Later we extended our research field to affective disorders, Tourette syndrome and at last, we turned our attention to the investigation of MPAs in epilepsy, on which we published our results in 2022, on the 25^th^ anniversary of our research work (**Table 2**).

**Table 2:** List of our working group’s research articles on minor physical anomalies with the use of the Méhes Scale.

No.	Article title	Year of publication	Authors and Journal	Main results
1	Informative morphogenetic variants in patients with schizophrenia and alcohol-dependent patient: beyond the Waldrop Scale	1997	Trixler M, Tényi T, Csábi Gy, Szabó G, Méhes K *Am J Psychiatry*	Schizophrenia patients had higher number of 3 MMs and 2 PVs, 4 of them were in the head region.
2	Problems with the Waldrop Scale	2000	Trixler M, Tényi T *Am J Psychiatry*	Letter to the Editor. We suggest the need for distinguishing MMs and PVs on a new extended scale of dysmorphology
3	Minor physical anomalies in schizophrenia and bipolar affective disorder	2001	Trixler M, Tényi T, Csábi Gy, Szabó R *Schizophr Res*	Patients with schizophrenia had higher rates of three MMs malformations and one PV
4	Minor physical anomalies in non-familial unipolar major depression	2004	Tényi T, Trixler M, Csábi Gy, Jeges S *J Affect Disord*	There was no difference between the patient group and healthy controls in terms of MPAs.
5	Minor physical anomalies and chromosomal fragility as potential markers in schizophrenia. Preliminary report.	2005	Trixler M, Tényi T, Kosztolányi Gy *Int J Hum Genet*	Chromosomal fragility was increased in MPA positive patients with schizophrenia
6	Minor physical anomalies in Tourette syndrome	2008	Csábi Gy, Gádoros J, Jeges S, Gyenge E, Trixler M, Tényi T *Eur J Psychiatry*	Patients with Tourette syndrome had higher prevalence of MPAs (form which 4 was MM, 3 was PV)
7	Minor physical anomalies in affective disorder. A review of literature	2009	Tényi T, Trixler M, Csábi Gy *J Affect Disord*	Review article. Data suggest a higher probability of the role of abnormal neurodevelopment in patients with bipolar disorder compared to unipolar depression.
8	Neurodevelopment and schizophrenia: data on minor physical anomalies and structural brain imaging	2011	Tényi T *Neuropsychopharmacol Hung*	Review article. Data on both MPAs and structural brain imaging results underlie the neurodevelopmental hypothesis of schizophrenia.
9	Minor physical anomalies in autism [Hungarian]	2013	Tényi T, Jeges S, Halmai T, Csábi Gy	Prevalence of MPAs was higher in the autism group.
10	Minor physical anomalies are more common in children with idiopathic epilepsy	2014	Csábi Gy, Zsuppán R, Jeges S, Tényi T *Neuropsychopharmacol Hung*	The mean value of total count of MPAs was higher in children with idiopathic epilepsy syndromes
11	Minor physical anomalies are more common in schizophrenia patients with the history of homicide	2015	Tényi T, Halmai T, Antal A, Benke B, Jeges S, Tényi D, Tóth ÁL, Csábi Gy *Psychiatry Res*	MPAs were more common in homicidal schizophrenia patients compared to non-homicidal schizophrenia patients
12	Minor physical anomalies are more common among the first-degree unaffected relatives of schizophrenia patients – results with the Méhes Scale	2016	Hajnal A, Csábi Gy, Herold R, Jeges S, Halmai T, Trixler D, Simon M, Tóth ÁL, Tényi T *Psychiatry Res*	PVs were more common among the first degree, unaffected relatives of patients with schizophrenia One MM (flat forehead) was more prevalent compared to controls
13	Iris structure and minor physical anomalies in schizophrenia	2017	Trixler D, Tényi T *Psychiatry Res*	There were significant differences in the frequency of iris patterns and MPAs between schizophrenia patients and controls
14	Minor physical anomalies in bipolar I and bipolar II disorder – results with the Méhes Scale	2017	Berecz H, Csábi Gy, Jeges S, Herold R, Simon M, Halmai T, Trixler D, Hajnal A, Tóth ÁL, Tényi T *Psychiatry Res*	Patients with both bipolar I and bipolar II disorders had higher number of MPAs, MMs and PVs, especially in the head region.
15	Minor physical anomalies and dermatoglyphic signs in affective disorders: a systematic review	2017	Berecz H, Csábi Gy, Herold R, Trixler D, Fekete J, Tényi T	Review article. The relative contribution of the role of abnormal neurodevelopment in affective disorders needs further clarification.
16	Minor physical anomalies in bipolar disorder – a meta-analysis	2021	Varga E, Hajnal A, Soós A, Hegyi P, Kovács D, Farkas N, Szebényi J, Mikó A, Herold R *Front Psychiatry*	Based on 4 studies with 155 patients, patients with bipolar disorder had higher rates of MPAs, especially in the head region.
17	Increased prevalence of minor physical anomalies among healthy first-degree relatives of bipolar I patients – results with the Méhes Scale	2021	Csulak T, Csábi Gy, Herold R, Vörös V, Jeges S, Hajnal A, Kovács MÁ, Simon M, Herold R, Tóth ÁL, Tényi T *Front Psychiatry*	MPAs were of higher rate in the first-degree, unaffected relatives of patients with bipolar disorder
18	Increased prevalence of minor physical anomalies in patients with epilepsy	2022	Tényi D, Tényi T, Csábi Gy, Jeges S, Bóné B, Lőrincz K, Kovács N, Janszky J *Sci Rep*	Patients with epilepsy had higher prevalence of MPAs, MMs and PVs compared to controls in all epilepsy groups except for acquired epilepsy

MM, minor malformation; MPA, minor physical anomaly; PV, phenogenetic variant.

### 4.1 Schizophrenia

It has been argued that the dichotomist approach of the etiology of schizophrenia, that is the differentiation of neurodegenerative and neurodevelopmental mechanisms, is artificial and misguiding as the pathophysiology may show elements of both processes (12, 33–35). Magnetic resonance imaging studies indicated progressive changes in cerebral structures, fortifying the concept of neurodegeneration, however, the neurodevelopmental theory still remains as focal point. This hypothesis suggests that as a result of different insults (stress, obstetric complication, infection, vitamin D deficiency among others) occurring on grounds of a genetically defined susceptible constellation (12), subtle abnormalities in brain development occur *in utero*, remain latent for years before manifesting as symptoms of schizophrenia (36–39). Moreover, Catts and colleagues argue that neurodevelopment is not final at birth but continue postnatally well into adolescence, considering schizophrenia as a result of defected cortical maturation and retainment of an immature cerebral cortex (12). The neurodevelopmental theory of schizophrenia is fortified by the numerous imaging studies reporting on reduced gray matter volumes in the mesiotemporal structures and frontal lobe, enlarged lateral and third ventricles, abnormal gyrification patterns and heterotopic lesions (34, 40, 41).

Another strengthening factor of the neurodevelopmental theory is the excess of MPAs in patients with schizophrenia, which has been detected in several studies (18, 19, 28, 42–44). In the majority of them the Waldrop Scale or a modification thereof was used to assess MPAs (28, 29, 45, 46). The sometimes conflicting results of these studies may be, at least partly attributed to the scale in question itself, since it’s not comprehensive and unable to provide information on the timing of maldevelopment, which would be crucial in terms of future studies on possible primary/secondary prevention. With the use of the Méhes Scale, we detected five minor physical anomalies to be more common in patients with schizophrenia compared to patients with alcohol dependence serving as control subjects (47). As the Méhes Scale differentiates between minor malformations and phenogenetic variants, we were able to gain further insight regarding the timing of maldevelopment. We found that three of these MPAs were minor malformations (furrowed tongue, multiple buccal frenula, hemangioma), and the other two were phenogenetic variants (protruding auricle, large tongue), thus we can conclude that the insult is not just limited to a single developmental step but rather owns a long-acting effect and induces complex changes in neurodevelopment (47). In another study of ours, we have found that compared to normal controls, patients with schizophrenia showed higher rates of three minor malformations (furrowed tongue, flat occiput, primitive shape of ears) and one phenogenetic variant (wide distance between the toes 1 and 2). Also, as compared to patients with bipolar affective disorder, patients with schizophrenia had a higher number of one minor malformation (primitive shape of ears) (48). Given the comprehensive nature of the scale, we were able to study the topological profile of MPAs. We have found that they are mostly confined to the head and mouth region, fortifying their hypothesized connection to defective neurodevelopment (47, 48). In another study of ours we were the firsts to study iris structural patterns in schizophrenia patients in relation to MPAs. Our results fortified the neurodevelopmental concept by revealing a connection between structural iris patterns and MPAs (49).

It has been debated in what degree are MPAs results of epigenetic or genetic causes. There were studies that suggested a familial-genetic (20) background, on the other hand, the significant contributing role of epigenetic factors is supported by a study that found a higher prevalence of MPAs in patients with schizophrenia with a negative family history (50), however *de novo* genetic variants could also explain a large proportion of schizophrenia cases and be responsible for MPAs at the same time. In a study of ours we have found increased chromosomal fragility in MPA-positive patients with schizophrenia compared to controls, which provides significant support for the genetic determination of MPAs (51).

One of the most important clinical aspect of schizophrenia is the response rate to treatment, hence the overall severity of the disease. Treatment resistance has been connected to an increased prevalence of MPAs, which implies stronger insults in early neurodevelopment, thus, a more pronounced neurodevelopmental effect in pathogenesis (52). This result is in line with neuroimaging studies: treatment resistance has been connected to more reduced gray matter volume in frontal areas, enlarged white matter volume based on structural MRI studies, and different connectivity in resting state MRI studies (53). Another major clinical feature of schizophrenia is the risk of violence and aggressive behavior of the patients, which could be regarded as a factor of disease severity. The important role of aberrant neurodevelopment in the degree of violence in patients with schizophrenia has been supported by neuroimaging studies, detecting more pronounced frontal and temporal lobe abnormalities (54). Crowner and colleagues were the firsts to investigate the frequency of MPAs in violent psychiatric patients. The study was conducted using the Waldrop Scale and detected no relationship between MPA profile and violent behavior (55). Applying the Méhes Scale on a homologous sample, examining schizophrenia patients with the history homicide, we have found a clear positive correlation between the MPA frequency and violent behavior, implying a more severely affected neurodevelopment in these patients (56). According to the results of an electrophysiology study, having been conducted with the use of a modified version of the Méhes Scale, a possible endophenotype could be the abnormal profile of gamma oscillations, which are detectable with quantitative electroencephalogram (EEG) in schizophrenia patients. This was the first study on gamma activity in relation to MPAs in schizophrenia, however the authors argue that gamma abnormalities are well known phenomena in schizophrenia. Increased gamma power and synchrony are correlated with positive symptoms, consistent with enhanced structural connectivity, on the other hand, negative symptoms have been correlated in both increased and decreased activity (57). We consider these results to be beneficial not only in terms of research in schizophrenia etiology but also for clinical practice, since it may serve as a screening tool for therapy resistance and possible violent behavior.

### 4.2 Affective disorders

Since the early 1900s bipolar disorder (formerly known as “manic-depressive insanity”) has been carefully distinguished from other psychotic disorders (58). Since then, there is a growing evidence that the clear distinction of these disorders might be misleading, and the new concept of a psychiatric continuum ranging from unipolar depression, bipolar disorder, schizoaffective disorder and schizophrenia has been introduced (58–60). The theory of this continuum, more precisely the connection between bipolar disorder and schizophrenia has been strongly supported by decades-long research as these two disorders share many characteristics. A certain level of genetic overlap has been detected, they have several common risk factors, various structural brain abnormalities, share endophenotypes (neurocognitive, neuropsychological impairment, interpersonal difficulties) and regarding the pathophysiology, neurotransmitter abnormalities are thought to play a central role in both disorders. Based on this great amount of evidence, the theory of aberrant neurodevelopment as a central etiological factor could be extended from schizophrenia to bipolar disorder (58, 59, 61, 62).

Although it has been ongoing since the early 1990s, the strength of evidence from research of MPAs in affective disorders is not high. It can be traced back to the rather small amount of studies with conflicting results, which can be attributable to the – in many cases – heterogenous patient groups (mixed groups of bipolar, unipolar and schizoaffective patients) and the use of the Waldrop Scale or a modified version thereof (63, 64). In those few studies where patients with unipolar and bipolar depression were analyzed separately, results seem to be inconsistent: in some of them an increased frequency of MPAs in patients group could be detected compared to controls, however, these results could not be replicated in other studies, reviewed by Tényi et al. (63).

Acknowledging the rising concept of an existing continuum between unipolar depression and schizophrenia, we decided to extend our research on MPAs from schizophrenia to affective disorders and to try to resolve the conflicting results with the use of the Méhes Scale. In our first study (48), we have found that one minor malformation, namely furrowed tongue was more common in patients with bipolar disorder. In another, more extended investigation, we analyzed patients with bipolar I and bipolar II disorder separately. We detected an increased frequency of the total number of MPAs, minor malformations and phenogenetic variants in both bipolar I and bipolar II patient groups compared to controls. Moreover, analyzing the total number of anomalies in the mouth and ear region, we have found a higher frequency in patients with both bipolar I and bipolar II disorders. There proved to be no difference between the two patient groups in terms of the total number of MPAs, minor malformations and phenogenetic variants. In course of the individual analysis of the 57 MPAs, we have found that furrowed tongue is more common in bipolar I disorder and high arched palate is more common in patients with bipolar I and II disorder (65). On the other hand, in another study of ours, analyzing patients with non-familial unipolar recurrent major depression, we detected no difference between depressive patients and controls neither in the total number of MPAs, nor in the individual analysis of each anomaly (66). Conducted by our research group, in a recent meta-analysis, including four studies with 155 patients, an increased number of MPAs could be detected in patients with bipolar disorder compared to healthy controls (62). Taken together, our studies on the MPA profile of affective disorders underlie the current concept of the disease continuum between affective disorders and schizophrenia and the consequent rise of the theory of abnormal neurodevelopment being an etiological factor in bipolar disorder. Similar to schizophrenia, in terms of the topological profile, a definite head-area dominance of MPAs can be observed, further supporting neurodevelopmental origin. Distinguishing minor malformations from phenogenetic variants we gained further insight into the dynamic of maldevelopment: having detected that both minor malformations and phenogenetic variants are more common is bipolar disorder – similar to schizophrenia –, it implies a longer interval of CNS vulnerability to insults predisposing to bipolar disorder.

### 4.3 Analysis of MPAs in unaffected relatives of schizophrenia and bipolar patients

Endophenotypes are heritable traits that are indicators for a genetic susceptibility of a psychiatric disorder, they bridge the gap between symptom presentation and genetic variability. According Gottesman and Gould (67) “Endophenotype should be (1) associated with the illness, (2) heritable, (3) state-independent, (4) found in unaffected relatives at a higher rate than in the general population, and (5) shown to co-segregate with the illness within families” (68, pp 225). Since both schizophrenia and bipolar disorder operate with a strong genetic background, the intensive research on endophenotypes are by all means justified. Reviewing the analyses on the MPA profile of healthy relatives of schizophrenia patients, results seem to be conflicting: using the Waldrop Scale or modified Waldrop Scales, certain studies have found an increased number of MPAs in unaffected first-degree relatives, which results could not be replicated in other investigations (68). In our study on first-degree relatives of schizophrenia patients, with the use of the Méhes Scale, an overrepresentation of MPAs could be detected compared to controls, moreover, in line with our previous results, these anomalies had the tendency to appear in the mouth and head regions (68). More recently we turned our attention and extended endophenotype research towards affective disorders. We were the firsts to report on an increased number of MPAs among the first-degree relatives of patients with bipolar I disorder (69). Since in the individual analyses, among the two overrepresented MPAs, one was a minor malformation (sole crease) and the other one was a phenogenetic variant (high arched palate), it seems plausible that aberrant neurodevelopment may appear anytime between 3^rd^ and 20-23^rd^ gestational weeks (69). Increased number of MPAs in the unaffected relatives support the hypothesis about MPAs serving as endophenotypic trait markers in schizophrenia and bipolar I disorder, which, apart from its scientific value in etiology research, may also hold promise in prevention.

### 4.4 Tourette syndrome

Tourette syndrome is a neuropsychiatric syndrome characterized by multiple chronic tics, that may be either simplex or complex, vocal or motor. Its etiology seems to be multifactorial. Tourette syndrome is considered to lie on genetic grounds, being polygenic, involving several common risk variants mixed with uncommon, inherited or *de novo* genetic mutations. Besides the genetics, epigenetic factors may also contribute to the pathogenesis, resulting in structural and functional brain anomalies (70). Growing evidence suggests the strong neurodevelopmental nature of the disease. Supplying parallel support for the structural imaging studies, we were the firsts to investigate the MPA profile of children with Tourette syndrome. We detected a higher number of MPAs in the patients group compared to healthy controls and, similarly to our other studies on schizophrenia and bipolar disorder, these anomalies were more pronounced on the head region (high arched palate, posterior hair whorl), further supporting the neurodevelopmental theory (71).

### 4.5 Autism spectrum disorder

Autism, a disorder with the core symptoms of communication deficit, impaired social interaction and repetitive, stereotypic behaviors and interests, is thought to be caused by a complex interplay between multiple genetic and environmental factors. As opposed to earlier beliefs about autism carrying a strong psychological aspect (e.g. refrigerator mother theory), decades-long research has proven that it is in fact a neurodevelopmental disorder and psychological factors hardly even contribute to the pathogenesis (72, 73). In support of this theory, data from seven studies on the frequency of MPAs in autism (which were conducted by the Waldrop Scale) were analyzed in a meta-analysis, which showed a higher prevalence of MPAs in autism. However, as there were only very few data on the individual analysis of MPAs in autism, we analyzed 20 patients with the use of the comprehensive Méhes Scale (74). Our results corresponded to the previous studies, since we detected an increased frequency of MPAs in patients compared to controls, moreover, in course of the individual sub-analyses, primitive shape of ears, abnormal philtrum, clinodactylia and wide distance between toes 1 and 2 have proven to be the most common (74). After our publication, a Swedish research group published a study - analyzing minor malformations and phenogenetic variants separately in a similar way to ours - in which they reported a significantly higher overall MPA prevalence among adult patients with autism spectrum disorder, moreover they have found a higher prevalence of MPAs in the craniofacial region, namely the ear (75).

### 4.6 Epilepsy

Epilepsy is a large heterogenic group of neurological disorders, differing in etiology. In many epilepsy syndromes, previously assumed to be caused by one specific anomaly (e.g. channelopathy in juvenile myoclonic epilepsy or hippocampal sclerosis in temporal lobe epilepsy), it has been recognized that abnormal neurodevelopment also contributes to the epileptogenesis, which resulted in the recent rise of the concept of epilepsy as a neurodevelopmental disorder (76).

Early in the 20^th^ century, increased frequency of MPAs in epilepsy patients was reported (77), however, these studies were not carried out with a scale based on modern dysmorphology. In a pilot study, analyzing 24 subjects and 24 controls, we detected an increased frequency of MPAs in patients with idiopathic childhood epilepsy (78).

We aimed to get further insight into the MPA profile of epilepsy patients, and in our subsequent study 235 adult epilepsy patients were included according to the following subgroups: acquired epilepsy (e.g. post-stroke, posttraumatic), temporal lobe epilepsy, epilepsy with cortical dysgenesis etiology, cryptogenic epilepsy and idiopathic generalized epilepsy (79). A higher number of MPAs in all epilepsy subgroups could be detected except for acquired epilepsy. The overrepresentation of these anomalies supports the view that epilepsy is related to factors early in development. However, in case of patients with acquired epilepsy, the insult seems to impact an intact nervous system, without a possible predisposing effect of a neurodevelopmental abnormality (79). In concordance with our studies on patients with schizophrenia, bipolar disorder, autism and Tourette syndrome, the two MPAs that were more common in epilepsy patients (furrowed tongue and high arched palate), both involved the head region (47, 48, 56, 65, 71, 74). Moreover, also similar to patients with schizophrenia, therapy resistance was associated with higher number of MPAs (52). Using the Méhes Scale as a screening tool, early identification of high-risk patients for pharmacoresistance may become possible.

## 5 Conclusion

Results of a wide range of decades-long research support the etiological role of aberrant neurodevelopment in disorders previously regarded as non-organic diseases. Besides genetic, epigenetic, neuroimaging and histological investigations, this growing evidence is further supported by the studying of MPAs, which serve as external markers for abnormal neurodevelopment. With the use of the Méhes Scale, a comprehensive, modern scale of dysmorphology, conducted by Professor Károly Méhes at the University of Pécs, our research group has been investigating the MPA profile of neurodevelopmental disorders since 1997.

25 years into our work we conducted research on patients with schizophrenia, bipolar disorder, unipolar depression, Tourette syndrome, autism and, on the 25^th^ anniversary of our research we published our results on MPAs in epilepsy patients, extending our research field from neuropsychiatric to neurologic disorders.

## In memoriam

In memoriam Professor Károly Méhes (1936-2007).
